# Perpetuating Commodification of Suffering: How Social Determinants of Health Framing Prolongs Historical Racial Inequities

**DOI:** 10.1089/whr.2021.0019

**Published:** 2022-03-04

**Authors:** Garssandra Presumey-Leblanc, Megan Sandel

**Affiliations:** ^1^ Department of Graduate Medical Sciences, Medical Anthropology and Cross-Cultural Practice, Boston University School of Medicine, Boston, Massachusetts, USA.; ^2^ Department of Pediatrics, Boston University School of Medicine, Boston, Massachusetts, USA.

**Keywords:** Social Determinants of Health, Commodification of Suffering, Black Maternal Health, Health & Human Services, Post Traumatic Slave Syndrome

## Abstract

**Background:**

In the United States, social determinants of health (SDOH) and the marginalization of Black and Indigenous people of color (BIPOC) populations often result in negative health outcomes, but may miss the underlying historical causes for these disparities. While the health and human services (HHS) workforce is trained to recognize and address negative SDOH, it is unclear how historical trauma impacts this contemporary work.

**Materials and Methods:**

As part of a broader ethnographic study, 2 MLP affiliates and 6 BIPOC women HHS workforce members who had taken SDOH trainings were interviewed using semi-structured interviews. Themes were explored around the language of SDOH and how the medical and legal system responses to SDOH results in negative health outcomes and historical trauma.

**Results:**

Themes emerged around American medical and legal institutions, meant to care for patients, have been wearied by the power struggle between politics, budgets, and the value of financial profit. Under such competing loads, workforce members feel trapped in financially-based hierarchies and established risk-designations. This current conceptualization of the language and coding of SDOH has created a globalization or commodification of suffering that mirrors historical hierachies in slavery.

**Conclusions:**

The variable naming of SDOH draws attention away from the root causes of inequities and the individualization of the social risks creates a cycle of commodification of suffering, instead of addressing the historical and structural antecedents. Future work should explore how to adapt to these intersections.

Social determinants of health (SDOH) help identify health inequities and systemic barriers experienced by black people in the United States. As Dr. Joia Crear-Perry et al. (2020) writes, SDOH has lost meaning within health care due to misuse and lack of context. Although the health care industry has acknowledged the SDOH, the constant renaming of this term has overshadowed its ability to explain black health outcomes.^1^ Most current frameworks do not explore the impact of historical trauma on contemporary experiences of SDOH. This warrants a deeper understanding of historical forces that underlie racist systems in the United States, including health care.

SDOH interventions, designed by the medical institution, are constrained by financial incentives and structural restrictions of the medical–legal institution that convolute the terminology used. Unfortunately, current interventions make the system efficient in its oppression of minority patients and workforce members, not reparative. This is commodification of suffering. To ameliorate negative health outcomes for black communities, the focus must be on implementing solutions that acknowledge historically oppressive antecedents, not terminology alone.

## Multiple Names, No Impact

Words such as “dimensions” have replaced “determinants”; “structural” has replaced “social.” Replacing “social” with “structural” acknowledges that institutional barriers precede social factors. There has been a differentiation between social determinants and social influencers of health based on determinism, inevitability, and permanence. This iteration aims to lessen the blame on individuals as proprietors and keepers of their health. Crear-Perry et al. (2020) agree with this shifting of accountability from individuals to systems. Therefore, maintaining “social” in SDOH does not acknowledge the structural impacts. Perhaps, a more appropriate phrase would be structural drivers of equity, replacing the word “health” entirely. Because the problem is not un-health, but in-equity.

The focus should be on identifying historical causes, not renaming SDOH. Naming SDOH should demonstrate the intersectionality of social, biological, and historical components. Variable naming of SDOH avoids challenging the medical–legal institution that contributes to systemic racism, and does not acknowledge how black populations experience negative SDOH due to that racism. There must be education that illustrates the link between historical discrimination and current negative health outcomes before instituting interventions to avoid replicating white dominant-driven solutions. The goal must be to relieve inequity and reflect on how current systems and interventions not only ignore history, but also perpetuate commodification of suffering.

## Commodification of Suffering: Risks and Accountability

Instead of renaming the term, we should refocus the conversation on the importance of structural determinants and “root causes of inequities.” Consistent with Crear-Perry et al. (2020) call to “elucidate the web of causation between structural and SDOH for black women and other disenfranchised groups has the potential to facilitate the identification of interventions and policies that can remediate and eliminate inequalities in health across the population”^1^ we must recognize that there are budgetary motives behind the current structure and investment in SDOH.

Insurance payers are establishing a financial hierarchy based on patient risk and suffering, with more money going to “higher risk” patients. It is apparent that risk is a major theme in the medical and legal realms. Patients’ risk designations warrant examinations into the profitability of illness.

It is apparent that risk was a major theme in the medical and legal realms. It has been made clear—the riskier the patient, the more profit for providers who screen for it. Despite the fact that riskier patients require more time and services, the influx of federal funds for SDOH allows for profit generation. This is exemplified through Boston Medical Center's THRIVE survey, one of many initiatives and potential solutions—a screening tool for negative SDOH vulnerability.

With time constraints hindering physicians who are limited to 15-minute visits, how are they to uncover nonmedical health-related issues? To ensure providers had the necessary tools of address this billable need, BMC launched the THRIVE Social Determinants of Health Screening and Referral Program to specifically identify not only needs in housing, food, and affording medications, but also in transportation, utilities, caregiving, employment, and education.

Patients can choose to fill out the screener while they wait for their provider.^6^ If the patient goes on to screen positive for any SDOH, they might be given a visit diagnosis or Z-code and offered the hospital's THRIVE resource referral guides. For example, a patient who is identified as having a transportation need, food insecurity, or unemployment will receive the visit diagnoses of *lack of access to transportation*, **Z91.89**, *lack of adequate food*, **Z59.4**, or *unemployed*, **Z56.0**, and orders might be placed in EPIC for *Social Determinant: Transportation*, *Social Determinant: Transportation Resource Guide*, *Social Determinant: Food*, *Social Determinant: Food Bank Referral*, and *Social Determinant: Employment*.

Although this benefits patients who now have their SDOH acknowledged, this commodification of social suffering has not guaranteed that the screen will produce results. Rather, this highlights the fact that payers such as Medicaid, Medicare, and insurance companies are more worried about diverting funds from transportation, infrastructure, and education rather than ensuring positive health outcomes for the disadvantaged.

Although the investment in SDOH was beneficial to the patients, the bottom line for those who determined SDOH designation was money. But why is there such a disparity between how much the United States spends on health care and the lack of positive health outcomes?

In *The American Healthcare Paradox: When Spending More Is Getting Us Less*, Elizabeth H. Bradley and Lauren A. Taylor conducted a comparative study, looking at health care data from thirty countries. Bradley and Taylor explore the narrow definitions of health care, unequal divisions between health and social services, and this country's aversion to government programs to effectively demonstrate how these components needlessly create suffering in individual lives, even as we continue to increase health care spending. This corresponds to a lack of investment into social and behavioral initiatives that would improve health metrics in black communities.

This system remains inextricably linked to slavery in the United States. Slavery's untold legacy is that the black body is a commodity. Black bodies have always been stolen from Africa—the bodies upon which systems, such as the health care institution, are built. Suffering is now incentivized in the medical–legal institution. The application of Z-codes to SDOH is one example of commodification of suffering. These codes insufficiently challenge the structural factors that negatively impact minority communities. The solution lies in actively dismantling oppressive institutions and being anti-racist.

## Financing Solutions for Negative SDOH: Dismantling or Perpetuating Institutional Inequities?

So, what is the solution? If we believe Bradley and Taylor, then the answer is federally subsidized social services.^3^ However, in a country that values neoliberal principles such as individual responsibility, society shames those who would ask for government-sponsored aid. In contrast, if investing in social services will cut costs in health care (the most expensive budget expenditure), then simply for financial convenience, why not do it? The cure lies in finding ways to intervene and emphasize respectful care that addresses the lived experiences of black communities. A new evidence base of cocreated solutions is needed to offset predominantly white-centered solutions to date.

To effectively complement the identification of SDOH, we must include contemporary training on interventions in black communities, emphasizing historical education and multilevel system examination. These improvements will serve as the foundation to ensure that underserved populations obtain appropriate social and health services. Without these changes, these institutions’ discriminatory foundations will continue to perpetuate current inequities in health care.

## Conclusion

SDOH may generate profit because its acknowledgment does improve health. Medical professionals have lobbied government and insurance companies to make nonmedical intervention billable. Skeptically, others believe that the medical and legal establishments have diagnosed and labeled SDOH as diseases for the purpose of generating revenue. In essence, these systems have capitalized on the populations’ lack of resources. As particular populations experience negative SDOH more than others, this diagnosed social suffering “becomes embodied in a particular life trajectory, environed in a concrete life world.”^4^

For black populations, structural violence is evident, as negative SDOH lead to lived experiences of poor health outcomes for which the person, the state, or the insurance company are then billed. Maybe the reason that the medical and legal institutions (excluding safety-net hospitals and professionals such as public interest attorneys) do not feel morally obligated to act on their patients’ behalf is because those in power are removed from these lived experiences.

The current conceptualization of the SDOH language has created a “globalization of suffering.” Arthur Kleinman calls this troubling, because “experience is being used as a commodity.”^5^ Unlike Kleinman's exploration into the commodification of suffering, the medical–industrial complex does not use moral sentiment to mobilize support for transformative action in SDOH. Instead, the government and insurers use monetary incentives to motivate providers to acknowledge this phenomenon. But what is really changing? The lives of the populations that experience negative SDOH are not improving. Perhaps the cure lies in finding ways to intervene and emphasize holistic patient care that addresses the lived experiences of the community being served.

In the era of #BlackLivesMatter and social unrest, we are finally considering how history has impacted communities of color. However, the necessary changes needed to improve the lives of the marginalized progress slowly. States paint black lives matter on their streets, but only one state has outlawed qualified immunity. Although the sentiments are appreciated, black people want to see real change and the slow rate at which society is responding demonstrates how the American institutions continue to be complicit in the oppression and devaluing of black lives.

This is evident in the Trump administration's handling of the coronavirus disease 2019 (COVID-19) pandemic. Severe acute respiratory syndrome coronavirus 2 (SARS-CoV-2) exposed the disproportionate mortality rate affecting the black population and the lack of governmental response in aiding and protecting these communities, which is not surprising, as this population is medically underserved and resource limited.

Here is where history becomes important. What slavery taught us is that the black body is a commodity. In the past, our bodies were the basis of the American economy and now, it is our money. The black dollar is a powerful tool in redesigning the system and yet, the system continues to murder with impunity—law enforcement and health care especially. Perhaps that is why the white majority is afraid and protestors are met with unparalleled vengeance and anger.

The stereotypes of a terroristic American history play a role in how the health care system identifies and treats black bodies contemporarily. In medicine, black bodies are believed to experience less pain and looked down upon. Black slave women should be credited for the innovation and ingenuity of obstetrics and gynecology, despite the fact that black women today are approximately four times more likely to die during labor and/or delivery. Black bodies have always been ripe for the picking—stolen from Africa; the bodies upon which systems such as the health care institution are built. Although organizations such as MLPB and others strive to do better, we are missing the point.

The interventions of today cannot succeed if the horrific past of slavery and colonialization remains silenced and unacknowledged. The white lives of today benefitted from the privilege of the colonial hierarchy and law enforcement was only created to catch slaves. Ensuring that all lives matter would require recognizing black people and other marginalized groups who have been oppressed for centuries. Dismantling the systems would require active anti-racial decolonialization efforts, forcing the privileged majority to re-evaluate their profit off black bodies and their works.

How can the commodification of suffering be eliminated in a system designed to profit from marginalized suffering bodies? The evolving language of SDOH is merely redirecting our attention. If the medical and legal institutions were morally driven to treat the Health related social needs of their patients, then they would seek to truly understand the historical factors that have placed those vulnerable populations in their current positions. The capitalistic and neoliberal principles value financial gain, not the betterment of the population. To combat this, there must be an acknowledgment of the impact of historical injustice. Moving forward, the institutions should include restorative justice and reparative capitalism so that black populations have the same equitable—not simply equal—opportunities.

Disparate black maternal care is a prime example of commodification of suffering. What is not acknowledged enough is that “…racism kills. Whether through force, deprivation, or discrimination [racism] is a fundamental cause of disease and the strange but familiar root of racial health inequities.”^2^ Black women, irrespective of privilege, education, and wealth, are at higher risk of adverse or even fatal outcomes during pregnancy. The resulting interventions encourage the fee-for-service model, generating profit. Recognizing the impact of the historical legacy of slavery and colonialization on the medical institution is the first step on the equitable care journey, especially for black and minority birthing people.

The antecedents of contemporary injustices need to be appropriately addressed and connected to negative population health outcomes. Moving forward, institutions should include restorative justice and reparative capitalism, so black populations have equitable—not equal—opportunities. This can be achieved through building up and assessing historical competency in medical–legal education to address this historical legacy.

The evolving language of SDOH is distracting. If the medical and legal institutions were morally driven to treat the health-related social needs of their patients, then they would seek to understand the historical factors that have placed underserved populations in their current positions. The capitalistic and neoliberal value profit, not population betterment. To combat this, there must be acknowledgment of the impact of historical injustice. Today's interventions cannot succeed if the horrific past of slavery and colonialization remains silenced and unacknowledged.

## Abbreviations Used

COVID-19: coronavirus disease 2019
SARS-CoV-2: severe acute respiratory syndrome coronavirus 2
SDOH: social determinants of health
